# Geology and land use shape nitrogen and sulfur cycling groundwater microbial communities in Pacific Island aquifers

**DOI:** 10.1038/s43705-023-00261-5

**Published:** 2023-06-07

**Authors:** Sheree J. Watson, Cédric Arisdakessian, Maria Petelo, Kekuʻiapōiula Keliipuleole, Diamond K. Tachera, Brytne K. Okuhata, Henrietta Dulai, Kiana L. Frank

**Affiliations:** 1 University of Hawai’i at Mānoa, Pacific Biosciences Research Center, Honolulu, HI USA; 2 University of Hawai’i at Mānoa, Department of Information and Computer Sciences, Honolulu, HI USA; 3 University of Hawai’i at Mānoa, Marine Biology Graduate Program, Honolulu, HI USA; 4 University of Hawai’i at Mānoa, Department of Earth Sciences, Honolulu, HI USA

**Keywords:** Biogeochemistry, Water microbiology

## Abstract

Resource-constrained island populations have thrived in Hawai’i for over a millennium, but now face aggressive new challenges to fundamental resources, including the security and sustainability of water resources. Characterizing the microbial community in groundwater ecosystems is a powerful approach to infer changes from human impacts due to land management in hydrogeological complex aquifers. In this study, we investigate how geology and land management influence geochemistry, microbial diversity and metabolic functions. We sampled a total of 19 wells over 2-years across the Hualālai watershed of Kona, Hawai’i analyzing geochemistry, and microbial communities by 16S rRNA amplicon sequencing. Geochemical analysis revealed significantly higher sulfate along the northwest volcanic rift zone, and high nitrogen (N) correlated with high on-site sewage disposal systems (OSDS) density. A total of 12,973 Amplicon Sequence Variants (ASV) were identified in 220 samples, including 865 ASVs classified as putative N and sulfur (S) cyclers. The N and S cyclers were dominated by a putative S-oxidizer coupled to complete denitrification (*Acinetobacter*), significantly enriched up to 4-times comparatively amongst samples grouped by geochemistry. The significant presence of *Acinetobacter* infers the bioremediation potential of volcanic groundwater for microbial-driven coupled S-oxidation and denitrification providing an ecosystem service for island populations dependent upon groundwater aquifers.

## Introduction

Microbes play a regulatory role in the convoluted interactions between hydrology, geology, and land use [1, 2] resulting in highly variable groundwater biogeochemistry. Microbes in subsurface groundwater aquifers represent roughly 40% of Earth’s microbial life [3, 4], and are crucial for ecosystem services including providing clean drinking water. Subsurface microbial communities improve water quality by breaking down organic material and cycling nitrogen (N), sulfur (S), and iron (Fe) [5]. Unlike the redox structuring of sedimentary systems, opposing redox reactions co-occur in unique microenvironments (e.g., biofilms, particles) resulting in a diverse groundwater microbiome in large subsurface aquatic habitats [6, 7]. As a result, the use of inorganic compounds as energy sources (chemolithoautotrophy) are very common metabolic pathways in groundwater ecosystems, often linked to carbon (C), hydrogen (H), and sulfur (S) cycles with oxygen(O) and nitrate (NO_3_^−^) as the most widely used electron acceptors [1]. In one of the few microbiome studies conducted in deep Hawaiian groundwater aquifers, analysis revealed high functional diversity characterized primarily by chemolithotrophic metabolisms [8].

Characterizing the microbial community of groundwater ecosystems is a powerful approach to infer geochemical changes resulting from human impacts and land use in hydrogeologically complex aquifers [9, 10]. Changes in subsurface microbial community structure and diversity can alert users to decreases in water quality, because microbes in the subsurface react rapidly to alterations in their environment due to land use changes and/or contaminants, resulting in decreased diversity and shortened biogeochemical pathways [11, 12]. This is particularly important in Hawai’i as it depends almost entirely on groundwater for all water resources. Approximately 89% of Hawai’i’s potable water supply is located in subsurface water resources, with minimal contributions from surface water (i.e., reservoirs, rain catchment) [13], and small-scale desalination activities. Hawai’i has the greatest number of cesspools per capita in the United States [14]. Non-point source pollution from personal on-site sewage disposal systems (OSDS, cesspools) and agriculture are the two major sources of human-derived nutrients that impact coastal ecosystems and have been recognized as major environmental problems in Hawai’i [15–18].

Nitrogen is one of the most common nonpoint source contaminants in groundwater [12, 19, 20]. Inputs of N are important determinants of major ion groundwater geochemistry [21], and subsurface microbial communities are responsible for regulating the effects of anthropogenic N and its transition into surface water or coastal ecosystems [22–24]. Autotrophic denitrification is the dominant N-removal process in oligotrophic groundwater where inorganic compounds (Fe, S, manganese, Mn; hydrogen gas, H_2_) are oxidized by reducing nitrite (NO_2_^−^) or nitrate (NO_3_^−^) to gaseous nitrogen (N_2_) [25]. [23, 26]. In addition, microbial-driven denitrification can occur both anaerobically, which was thought to be the only process until the 1980’s, and aerobically, first described in a sulfide-oxidizing wastewater treatment plant [27, 28]. Human inputs of N into groundwater and its detrimental effects on coastal ecosystems has been heavily studied in Hawai’i [29–31], however the effect of excess N in volcanic, deep, freshwater aquifers and their subsurface microbial communities has yet to be characterized.

In addition to N, the presence of sulfate drives community structure in groundwater systems, and often points to a link between substrates and availability of electron donors [5, 32]. The presence of S species such as sulfate (SO_4_^2−^), sulfides (S^2−^), polysulfides (S_x_O_6_^2−^), thiosulfate (S_2_O_3_^2−^), and sulfite (SO_3_^2−^) are indicative of inputs from geothermal activity or seawater in groundwater systems [33–35]. The presence of SO_4_^2−^ in groundwater is often attributed to products of abiotic reactions, however, both anaerobic and aerobic biological sulfide oxidation is thermodynamically favorable in most environments [36]. Further, the relative availability of reduced S, C and Fe are also key determinants of nitrate removal pathways [26]. When S is present in carbon-limited systems, denitrification is powered by microbial mediated oxidation of reduced sulfur to sulfate [23], and occurs much faster than abiotic production of sulfate.

The Hualālai watershed (Kona, Hawai’i) has no surface runoff or drainage [37], is a semi-arid climate and is acutely vulnerable to stress from urban development due to the complete dependence on groundwater for municipal and agricultural water use [38]. Further, economic demands to support development in the Keauhou aquifer seek to exploit these aquifers and put undue strain on the quality of groundwater supplies. The watershed has the potential for large inputs of N due to personal OSDS, as well as S inputs from salt-water intrusion, aerosol deposition, and geothermal activity. In addition, fluctuating rainfall patterns reveal an overall drying trend, and rising temperatures is predicted to result in decreasing recharge and groundwater storage threatening the future of secure and safe freshwater supplies [39, 40].

In this study, we investigated subsurface microbial community structure and function with regard to N and S cycling in deep, volcanic Hawaiian island aquifers. We explored 1) how geology and land management practices in the Hualālai watershed influenced aquifer geochemistry, 2) the factors driving variability in microbial communities including geothermal inputs (S) along the rift zone and OSDS (N) inputs in the southern Keauhou aquifer, and 3) the microbial functional capacity for N and S cycling and how it varies with geochemical spatial pattern in these aquifers. The large and complex groundwater volcanic aquifers in Hawaii provide an opportunity to characterize the N and S cycling functions of microbial communities across different geologies and land management practices. This research has implications for land and water managers that must address decreased water quality in these drinking water resources affecting public health and future development in this watershed.

## Materials and methods

### Site description: Hualālai watershed

**Fig. 1:**
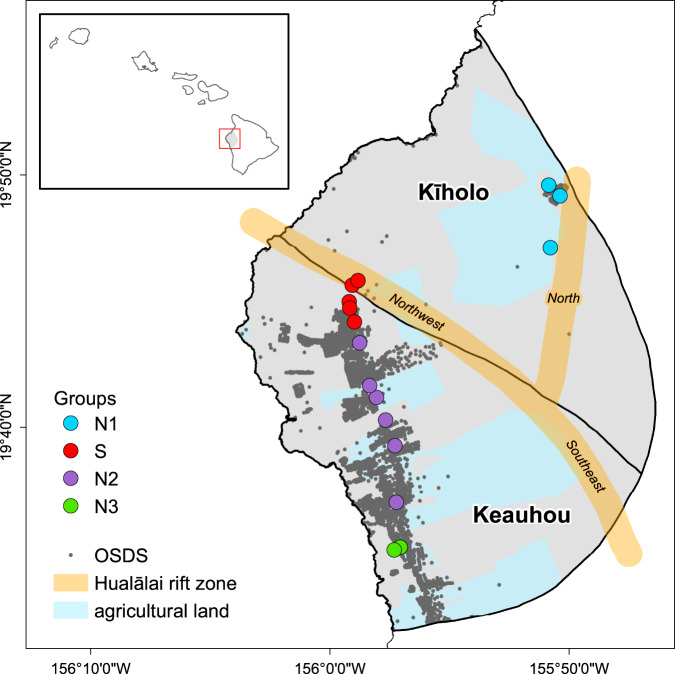
A map of the 19 sample sites in the Kīholo and Keauhou aquifers of the Hualālai watershed (colored circles). Sample sites are colored by groups (N1, S, N2, and N3), and the Hualālai rift zones are named by direction (North, Northwest, and Southeast). Agriculture lands are shaded light blue, and OSDS sites are indicated by black circles.

Hawai’i Island is the largest (10,464 km^2^) and youngest island in the Hawaiian archipelago [41]. The island was formed by five shield volcanoes with overlapping spatial and temporal lava flows, and geothermally active rift zones, which created complex hydrogeological aquifer connections [42, 43]. Hualālai, with a peak elevation of 2500 m above mean sea level, is the third most active volcano and consists of the Kīholo and Keauhou aquifer systems [44, 45]. Basalt flows and cinder cones build Hualālai and lie primarily along three geothermally-active rift zones trending northwest, northeast and southeast from the volcano summit (Fig. 1) [46]. The Hualālai watershed is located on the leeward (dry) side of Hawai’i Island with annual rainfall along the Kona coast ranging from 204 to 750 mm, however, the Kona rain belt upslope receives higher annual rainfall between 750 and 1350 mm [47].

### Sample collection

Groundwater samples were collected from 19 private and publicly owned vertical or inclined shaft production wells in quarterly intervals from August 2017 to March 2019 (Fig. 1; Supplemental Information (SI)). Raw groundwater samples were collected prior to chlorination after sample rinsing 10-L cubitainers 3 times at well pump stations following a 15–20 min well purge [48] and stored at 4 °C until arrival in the field laboratory. Groundwater was subsampled into acid-washed 250-mL HDPE (Nalgene) bottles and stored (15–30 days) at 4 °C until geochemical analysis. Triplicate 2-L samples were filtered first through a 47-mm diameter 0.8 µm (GH Polypro, Pall Gelman Inc., MI) and then through a 0.2 µm pore-sized hydrophilic polypropylene membrane (Pall Gelman Inc., MI) filter and both were frozen at −20 °C prior to DNA extraction.

### Geochemical analysis

Groundwater was separated into acid-washed 250 mL HDPE (Nalgene) bottles and stored (15–30 days) at 4 °C until nutrient analysis. Standard methods for nutrient analysis were followed according to manufacture protocols for a four channel autoanalyzer (Astoria-Pacific International, Astoria Pacific, Clackamas, OR). Total N and P was analyzed as PO_4_^3−^ and NO_x_^−^ following alkaline persulfate digestion [49]. Nutrient concentrations were determined for NO_3_^−^ + NO_2_^−^ = NO_x_^−^; orthophosphate, (PO_4_^3−^); silicate (Si); ammonium (NH_4_^+^); total nitrogen, (TN); and total phosphorus, (TP). A YSI Pro Plus multiparameter water quality meter was used to measure groundwater temperature (°C), pH, dissolved oxygen (DO, mgL^−1^), and specific conductance (SPC, µS cm^−1^) after pump flushing and prior to the collection of water for DNA analysis (YSI, Inc., Yellow Springs, OH). Samples for major ions (SO_4_^2−^; magnesium, Mg^2+^; chloride, Cl^−^) and select trace metals (chromium, Cr; iron, Fe; and manganese, Mn) were filtered through a 47-mm, 0.2 µm pore-sized hydrophilic polypropylene membrane (Pall Gelman Inc., Ann Arbor, MI) in a reusable filter holder and receiver (Thermo Fisher Scientific, Waltham, MA) and separated into 60-mL HDPE acid-washed bottles. Further information regarding geochemical analysis is provided in Supplementary Information.

### GIS land use metadata

Geographic spatial land use data was analyzed using the sf package [50] for R software to perform spatial analysis. Sample sites were converted to vector data as a 1 km circle around each site and land use data was aggregated and compared by site. Land use types include probability of high temperature (inferred caldera boundary/geothermal activity), agriculture (cropland and pasture, orchards, vineyards, ornamental horticulture), land use planning and allocation (urban high human density, rural human density, conserved lands), acres as golf courses, and resorts, as well as quantity of OSDS and total effluent flux in millions gallons per day [51].

### DNA extraction and 16S rRNA Illumina sequencing

DNA was extracted from all filters according to the PowerWater DNA Extraction protocol using the DNeasy PowerWater Kit (QIAGEN, Germantown, MD). Library preparation for 16S rRNA gene sequencing was performed in 4 separate libraries due to the length of the study and quarterly sampling between 2018 and 2019 using dual-indexed primers for the MiSeq Illumina platform by protocols described in Supplementary Information [52, 53]. Sequencing was performed on an Illumina MiSeq (300 cycle, V3 chemistry kit) at the UCI Genomics High-Throughput Facility, at U of California, Irvine. Methods for Quantitative PCR (qPCR) analysis of 16S rRNA, *dsrA* and *nirS* genes are provided in Supplementary Information.

### Bioinformatic and statistical analysis

Sequence data was processed through MetaFlow|mics pipeline [54, 55]. The dada2 R package [56] was used to filter, denoise and merge the raw reads into Amplicon Sequence Variants (ASVs). Reads were truncated at positions 250F and 170R and discarded if they contained one or more bases with quality scores <2 or >3. Sequencing error probabilities were modeled with dada2’s iterative learn errors function and denoised with an iterative partitioning algorithm with default parameters. Reads were merged and any pairs with an overlap of fewer than 20 bases, or with more than one mismatch, were discarded. Both the mothur v1.44.1 [57] and the Silva database (version 138) [58] were used to align and annotate sequences. Sequences with a start or stop position outside the 5th-95th percentile range (over all sequences) were discarded. Potential chimeras were removed with VSEARCH [59] as implemented in Mothur and assigned taxonomy using the RDP classifier [60]. ASVs with no taxonomic information at phylum level, or matching mitochondria or chloroplasts were discarded. Sequences can be referenced at BioProject ID: PRJNA819449. Statistical analysis was performed using the Phyloseq [61], Vegan [62], DESeq2 [63] factoextra [64], sf [50], and base statistical packages for R [65]. Nutrient and major ion concentrations were z-score transformed (mg L^−1^) prior to Principal Component Analysis (PCA). ASVs were subsampled to 2000 for Beta diversity (CCA, NMDS, PERMANOVA) analysis, and PERMANOVA tests were performed with 99999 permutations. An initial beta-diversity analysis was performed comparing the 0.8 and 0.2 µm 16S community and no significant difference (Permanova *p* > 0.5) was observed and therefore samples were combined for further analysis.

Putative functional annotations based on 16S rRNA genes were made utilizing version 1.2.4 of FAPROTAX (Functional Annotation of Prokaryotic Taxa; [66]. Due to recent taxonomic updates in the classification of certain clades of microorganisms [67] that are reflected in the Silva database (Silva138) but not in the FAPROTAX database, we manually re-annotated the FAPROTAX taxa of putative N and S cyclers to match Silva138 using the Genome Taxonomy Database (GTDB). A table of these taxa reclassifications along with their associated metabolisms are provided in Supplementary Information.

Differential abundance analysis (DESeq2) was designed for gene-level expression analysis of RNA-seq data, but is adapted and recommended for detection of differentially abundant species in high-throughput data as utilized in our analysis [68]. Differential abundance testing was performed on genera with N and S cycling capabilities with ASV prevalence greater than 3. A pseudo count of 1 was used to prevent null geometric means in the normalized process. *P*-values were computed using a Wald test and multiple hypothesis testing was adjusted with the Benjamini–Hochberg method [69]. Differentially abundant ASVs were extracted from all pairwise group comparisons, and selected for display in the heat map when the adjusted p-value was below 0.1 and the log2 fold-change above 1. The abundance values displayed were summed for each site, then converted to relative abundance. To improve heat map visualization, relative abundances values were converted to z-scores per ASVs. A list of displayed ASVs from DESeq2 analysis is provided in the SI.

## Results

### Physico-chemical groundwater characteristics

**Fig. 2:**
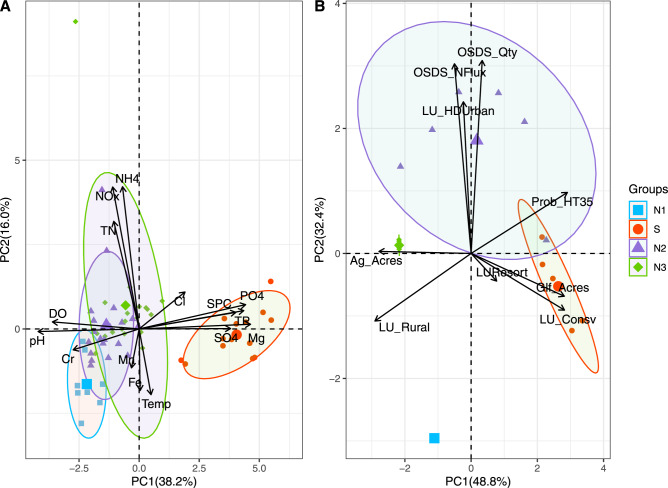
A principal component analysis (PCA) of associations between variables across sample groups. A principal component analysis (PCA) demonstrating associations between groundwater and **A** geochemistry variables, and **B** land-use, and geology. Summary variables are colored by sample groups (N1-square, S-circle, N2-triangle, and N3-diamond) with the larger shape representing the mean of principle variables, and black vectors representing strength of association inferred from vector length. Geochemistry includes pH; Cr Chromium, DO dissolved oxygen, NO_x_ nitrate+nitrite, TN total nitrogen, NH_4_ ammonium, Mn Manganese, Fe Iron, Temp temperature, Cl Chlorine, SPC specific conductance, PO_4_ orthophosphate, TP total orthophosphate, SO_4_ sulfate, Mg Magnesium. Land use includes conservation lands (LU_Consv), agriculture (Ag_Acres), high dentistry urban (LU_HDUrban), rural (LU_Rural), golf courses (Glf_Acres), resorts (LUResorts), OSDS quantities (OSDS_Qty), and OSDS N flux (OSDS_NFlux), and 35% probability of geothermal activity (Prob_HT35).

Based on principal component analysis (PCA), groundwater geochemistry across sampling sites clustered into two distinct groupings (PCA, Fig. 2A). Samples were categorized into an S group including five wells along the Hualālai rift zone (Fig. 1), and an N group consisting of 11 wells. Magnesium was the dominant driver (PC1 - 38.2%) of the differences between groups, followed by ammonium (NH_4_^+^; PC2 - 16.0%), which together explained 54.2% of the variation observed in geochemistry. The N groupings were further differentiated based on surrounding land use classifications. A PCA based on land use revealed four distinct groups explaining 81.2% of the observed variation (Fig. 2B). Group S formed a unique cluster based on an area with high probability of geothermal influence, while samples from group N were further clustered into N1 (rural land use), N2 (urban and OSDS), and N3 groups (agriculture; Fig. 2B). PCA revealed OSDS quantities were the dominant driver (PC1 - 48.8%) of variability in samples followed by conserved lands (PC2 - 32.4%).

**Fig. 3:**
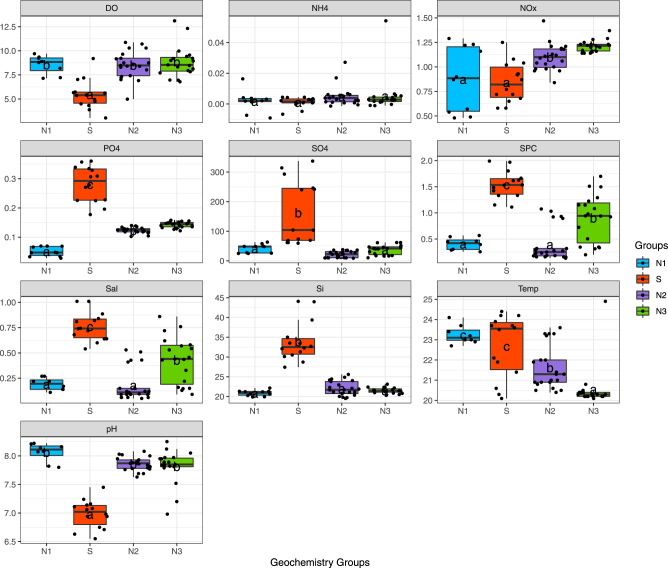
Groundwater geochemistry organized by groups (N1, S, N2, N3). The Mean (horizontal line), and individual sample values (dark circles) are indicated for each variable by group. Vertical y-axis for DO, NH_4_, NO_x_, and SO_4_^−2^, PO_4_^3−^, and Si are measured in mg L^−1^, SPC (µS cm^−1^), and Temp (°C). Post-hoc analysis (Tukey-post hoc) was performed to determine significant differences between groups and are labeled by letters to indicate significant differences.

Geochemistry of the S group was significantly different from N2 and N3 (Tukey, *p* < 0.001) for DO, NO_x_^−^, SO_4_^−2^, PO_4_^−3^, Si, SPC, salinity, and pH (Fig. 3). Group S was characterized by a significantly higher mean, and variable sulfate concentrations (179.02 mg L^−1^; Fig. 3). Several samples from one well in group S had SO_4_^2−^ concentrations greater than the EPA secondary maximum contaminant level threshold based on taste considerations (250 mg L^−1)^ [70]. The S group had lower mean concentrations of DO (5.03 mg L^−1^), NO_x_^−^ (0.88 mg L^−1^), pH (6.93), and high mean SPC (1.57 mS cm^−1^). In addition, mean averages of PO_4_^3−^ (0.29 mg L^−1^) and Si (33.91 mg L^−1^) were significantly higher for group S. In comparison, NO_x_^−^ was significantly higher in N2 and N3 (Tukey, *p* < 0.001) than group S. Samples from N3 had the highest mean concentrations of NO_x_^−^ (1.21 mg L^−1^) but are not significantly different from N2 (1.06 mg L^−1^). Two outlier samples of NO_x_^−^ occurred from N2 (5.73 mg L^−1^) and N3 (8.28 mg L^−1^), but were still below the EPA maximum contaminant level (mcl) for nitrates in groundwater (10 mg L^−1^) [21]. Groups in N also have significantly lower SPC (Tukey, *p* < 0.001), and higher pH (Tukey, *p* < 0.001; Fig. 3). Significant differences in geochemistry were not observed for sample times (months/year; Supplementary Fig. 1). Water quality parameters are published and available at the Hydroshare database [71].

A mantel test revealed significant spatial relationships between geochemistry and agriculture, urban density, golf course, and resort land use types (Table 1). Geochemical variables including DO (*R*^2^ = 0.66, *p* = 0.001), pH (*R*^2^ = 0.41, *p* < 0.05), SPC (*R*^2^ = 0.31, *p* = 0.01), and SO_4_^−2^ (*R*^2^ = 0.90, *p* = 0.001) were all strongly correlated with golf course and resort land use. The S group has the largest average land use as golf course and resort lands (1679 km^2^) and areas of high geothermal probability (Fig. 1). In contrast, NO_x_^−^ concentrations were strongly correlated with agricultural lands (*R*^2^ = 0.45, *p* < 0.01), and urban density (*R*^2^ = 0.32, *p* < 0.01; Table 1). The greatest average acreage (5160 km^2^) classified as urban occurred in group N3, while agricultural land use (pasture, conserved land) was greatest in group S and N1 with an average of 1.1 × 10^6^ km^2^. Mean quantities of OSDS were greatest in N2 at 5146 units per parcel, compared with 4009 in N3, and the smallest quantities occurring in S at 143.7 units per parcel. Rural land use was greatest by far in group S at 7080 km^2^ by comparison to all N groups with an average size of 1597 km^2^.

**Table 1:** Mantel test results to analyzed for spatial correlation between microbial community (ASVs), geochemistry (DO, SO_4_^−2^, and NO_x_ mg L^−1^, SPC µS cm^−1^, and pH), and land use management (OSDS, Agriculture, Urban density, Golf course and Resort lands, Rural density, and Conservation lands).

Variables	ASV’s	DO (mg L^−1^)	pH	SPC (µS cm^−1^)	SO_4_^2−^ (mg L^−1^)	NO_x_^−^ (mg L^−1^)
ASV’s	NA	*p* = 0.352 *R*^2^ = 0.045	*p* = 0.634 *R*^2^ = −0.083	*p* = 0.701 *R*^2^ = −0.051	*p* = 0.353 *R*^2^ = 0.048	*p* = 0.873 *R*^2^ = −0.169
OSDS	*p* = 0.013 *R*^2^ = 0.43	*p* = 0.117 *R*^2^ = 0.24	*p* = 0.089 *R*^2^ = 0.24	*p* = 0.045 *R*^2^ = 0.17	*p* = 0.137 *R*^2^ = 0.22	*p* = 0.146 *R*^2^ = 0.17
Agriculture	*p* = 0.626 *R*^2^ = −0.028	*p* = 0.016 *R*^2^ = 0.21	*p* = 0.011 *R*^2^ = 0.26	*p* = 0.15 *R*^2^ = 0.07	*p* = 0.324 *R*^2^ = 0.03	*p* = 0.004 *R*^2^ = 0.45
Urban density	*p* = 0.023 *R*^2^ = 0.20	*p* = 0.02 *R*^2^ = 0.21	*p* = 0.004 *R*^2^ = 0.32	*p* = 0.025 *R*^2^ = 0.22	*p* = 0.192 *R*^2^ = 0.05	*p* = 0.006 *R*^2^ = 0.32
Golf Course & Resorts - Rift zone	*p* = 0.54 *R*^2^ = −0.06	*p* = 0.001 *R*^2^ = 0.66	*p* = 0.016 *R*^2^ = 0.41	*p* = 0.003 *R*^2^ = 0.31	*p* = 0.001 *R*^2^ = 0.90	*p* = 0.302 *R*^2^ = 0.08
Rural density	*p* = 0.013 *R*^2^ = 0.32	*p* = 0.114 *R*^2^ = 0.17	*p* = 0.076 *R*^2^ = 0.21	*p* = 0.257 *R*^2^ = 0.04	*p* = 0.199 *R*^2^ = 0.02	*p* = 0.153 *R*^2^ = 0.19
Conservation	*p* = 0.019 *R*^2^ = 0.12	*p* = 0.026 *R*^2^ = 0.17	*p* = 0.044 *R*^2^ = 0.15	*p* = 0.213 *R*^2^ = 0.05	*p* = 0.264 *R*^2^ = 0.03	*p* = 0.021 *R*^2^ = 0.28

Shaded cells indicate strong correlation between variables. Units for land use include OSDS as quantities per parcel, and agriculture includes covered acres including pastures, farms, orchards, coffee, and floral. Urban, medium and rural density is based on census data for human populations. Resort and golf course land indicates acreage covered. Conservation is acres of lands in conservation status.

### Microbial community density and diversity

**Fig. 4:**
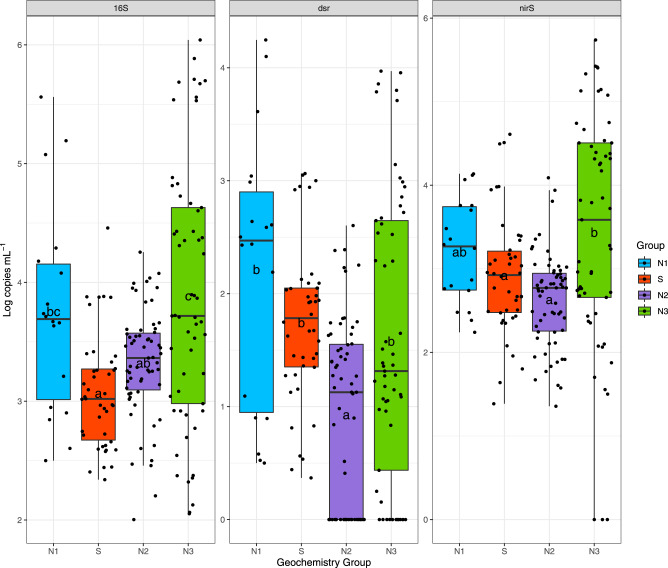
Quantitative PCR results for N and S metabolic genes (16S, dsrA, nirS) by groups in units of Log copies mL^−1^. Post-hoc analysis (Tukey-post hoc) was performed between groups and are labeled by letters to indicate significant difference.

Microbial density, as estimated from qPCR 16S rRNA gene copies mL^−1^, was low and significantly different across geochemistry groups (ANOVA, *p* < 0.001). The largest mean density was measured in N3 (9.7 × 10^4^ copies mL^−1^; Fig. 4) and the smallest abundances were measured in S (2.5 × 10^3^ copies mL^−1^). Group N3 had a significantly greater density than S (Tukey, *p* < 0.01), and N2 (Tukey, *p* < 0.001), but was not different from N1.

A total of 12,973 ASVs were identified in 220 samples collected between May 2017 and March 2019. Mean sample size was 12,966 reads per sample with a range of 2079–42,804. All samples were dominated by *Proteobacteria* (Supplementary Fig. 2) with mean relative abundances of 43.6% for *Gammaproteobacteria (Ɣ)* and 12.9% for *Alphaproteobacteria (α)*. The top 10 most abundant orders (Supplementary Fig. 2) made up >50% of overall relative abundances of groundwater samples. Beta-diversity of the microbial community was significantly different between PCA (S, N1, N2, N3) groups (PERMANOVA, *p* < 0.01; Supplementary Fig. 3). However, alpha-diversity (Pielou and Shannon indices) was not significantly different between PCA groups (Supplementary Fig. 4).

### Drivers of microbial diversity

**Fig. 5:**
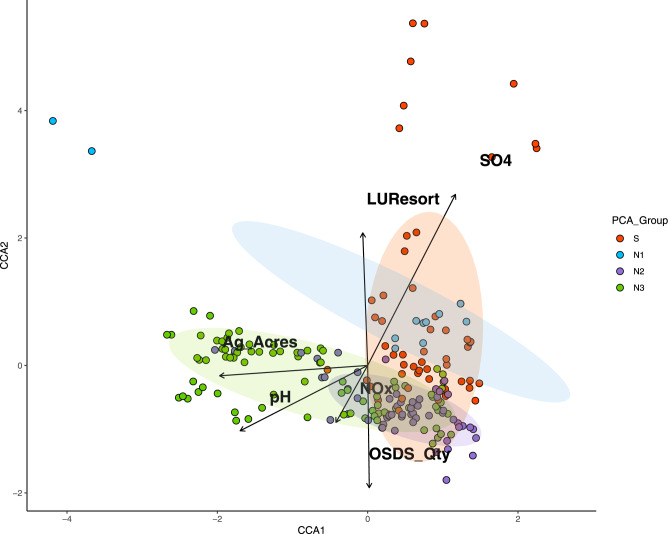
Canonical correspondence analysis (CCA) demonstrated associations between groundwater microbial community structure, geochemical variables, and land use management. Circles represent samples for each well colored by group, and black vectors represent association between communities, geochemistry and land use.

Canonical correspondence analysis (CCA) identified significant associations between groundwater microbial community structure, geochemistry, and land-use (ANOVA of CCA model, *p* < 0.001; Fig. 5). Agricultural land use (CCA1), pH, OSDS quantity, and NO_x_^−^ were significantly associated with microbial communities from groups N2 and N3, compared to resort land use and SO_4_^−2^ concentrations (CCA2) associations in groups N1 and S. Furthermore, mantel tests (Table 1) reveal that microbial communities were correlated with OSDS density (*R*^2^ = 0.43, *p* < 0.05), and rural densities (*R*^2^ = 0.32, *p* < 0.05). The mantel tests did not detect any significant correlation between community structure and agriculture, urban densities, rift zone, golf courses or resort lands, conservation lands, or any geochemical variable (Table 1).

### Functional capacity of microbial community

A total of 865 ASVs across groups (6.7% of total relative abundances) were classified as putative N and/or S cyclers between genus and order level based on Functional Annotation of Prokaryotic Taxa (FAPROTAX) [66]. Relative sequence abundances were calculated for each PCA group to account for differences in 16S rRNA gene abundances across groups.

**Fig. 6:**
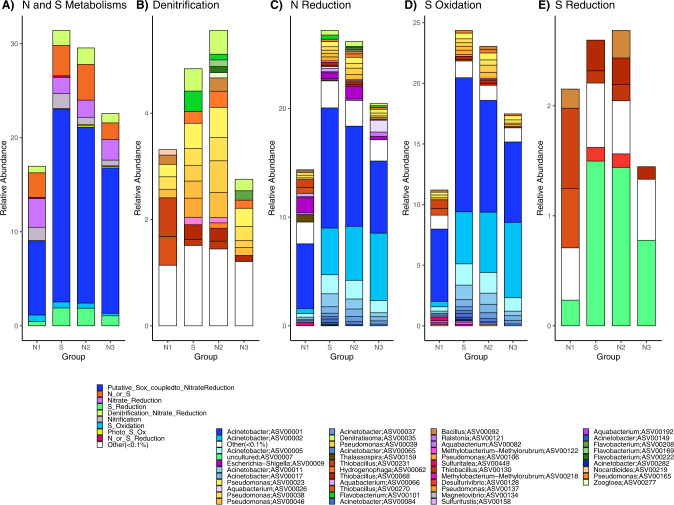
Relative abundance (%) of ASVs by PCA groups. Relative abundance (%) of ASVs are displayed by functional PCA groups including **A** N and S Metabolisms, **B** Denitrification included nitrate, nitrite, and nitrous oxide denitrification, **C** N Reduction includes N or S reduction, and nitrate reduction, **D** S oxidation includes photoautotrophic S oxidation, and **E** S Reduction.

Relative abundances of putative S-cyclers (353 ASVs) were significantly different across groups (*p* < 0.001; Fig. 6A). Group S had the highest mean relative abundances of putative S-cycling organisms (26.5%) and were significantly different from N1 (11.8%; Tukey, *p* < 0.01). Group N2 (25.2%) had significantly higher mean relative abundances of S-cyclers than N3 (18.8%; Tukey, *p* < 0.05) and N1 (Tukey, *p* = 0.0012). Putative S-oxidizers (159 ASVs) were dominated by the genus *Acinetobacter* with the highest mean relative abundances in group S (20.5%), compared with N2 (18.7%), N3 (15.5%), and lowest in N1 (7.9%; Fig. 6D). Sulfur reducers were dominated by an uncultured genus classified to order *Desulfobulbales* with low mean relative abundances (1.0%) across groups, and with the largest relative abundances occurring in group S (1.5%; Fig. 6E). Abundance of the sulfate reducing gene (*dsrA*) was also very low, but significantly different across all PCA groups (ANOVA, *p* < 0.001; Fig. 5). Group N1 had significantly higher mean abundances of *dsrA* (2.2 × 10^3^ copies mL^−1^) than N2 (*p* < 0.01). Group S (1.89 × 10^2^ copies mL^−1^) was also significantly different (*p* < 0.01) than N1 (2.15 × 10^3^ copies mL^−1^). If we assume an average of one *dsrA* gene copy per genome [72, 73], 7.7% of the microbial community of group S has the potential for S reduction, compared to 5.3% at N1, 1.1% at N2, and 0.94% at N3.

Putative N-cycling microbes (512 ASVs) also had significantly different relative abundances across groups (ANOVA, *p* < 0.001; Fig. 6A). Group S had the highest mean relative abundance of N-cyclers (28.9%), and was significantly different from N3 (20.91%; Tukey, *p* < 0.05) and N1 (15.27%; Tukey *p* < 0.01). Groups S and N1 had similar relative abundances of nitrifying organisms compared to lower abundances in groups N2 and N3. Most nitrifying microbes belonged to genus *Candidatus* Nitrosotenuis (Archaea) in N groups, compared to genus *Candidatus* Nitrotoga (Bacteria) in group S. Microorganisms capable of dissimilatory N reduction (via any part of the reduction cascade; 396 ASVs) had high mean relative abundances (18.5%) across all groups and were dominated by five ASVs of *Ɣ-proteobacteria* in genus *Acinetobacter*, with the largest relative abundance occurring in group S (11.1%; Fig. 6C). The next most abundant N reducer was classified to genus *Aquabacterium* with the highest relative abundance in group N3 (1.1%; Fig. 6C).

Denitrifying (capable of reducing nitrate, nitrite, or nitrous oxide to N_2_) microbes (212 ASVs) had low mean abundances (3.14%) and were not significantly different by groups. The largest denitrifying taxa contributions were from genus *Denitratasoma* with the largest relative abundance in group N2 (0.45%) and S (0.42%) and genus *Flavobacterium* in group S (0.38%; Fig. 6B). A more accurate measurement of denitrification potential measured by qPCR of the denitrifying gene (*nirS*) demonstrated high and significant differences between all groups (ANOVA, *p* < 0.001; Fig. 5). Group N3 had the highest mean *nirS* abundances (4.5 × 10^4^ copies mL^−1^), followed by S (3.88 × 10^3^ copies mL^−1^), and N1 (3.76 × 10^3^ copies mL^−1^) and N2 (1.0 × 10^3^ copies mL^−1^). The N3 group samples were significantly different from N2 (*p* < 0.001) and S (*p* < 0.01) for *nirS* gene abundances. Estimating an average one *nirS* gene copy per genome [74, 75] the majority of group S microbial community (>100%) has the potential for denitrification, compared to 9.3% at N1, 30.1% at N2 and 46.3% at N3.

### Differential abundance analysis (DESeq2)

**Fig. 7:**
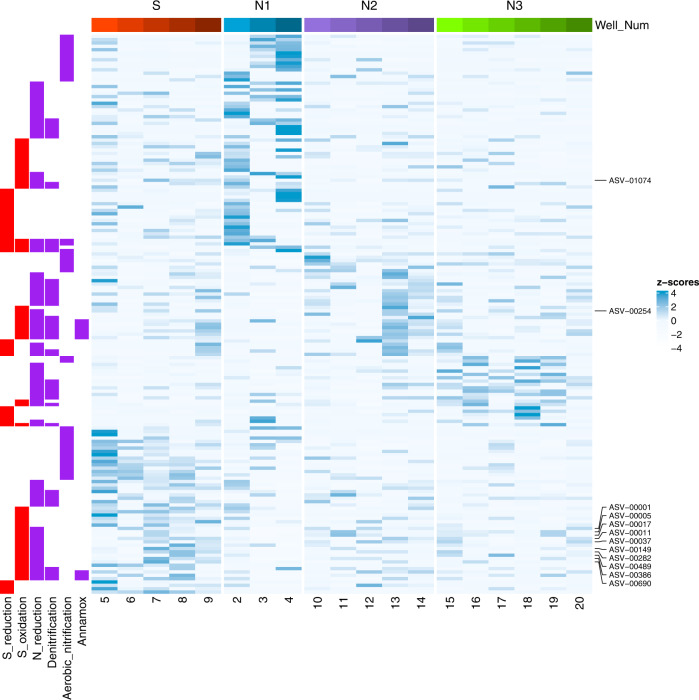
A heat map displays the abundance distribution of significantly different ASVs as designated by the DESeq2 model (adjusted *p*-value < 0.1 and a log2 fold-change >1). Abundance values for each ASV selected were summed by site and converted to relative abundance and transformed to z-scores for each ASV. ASV numbers identified as *Acinetobacter* are highlighted.

Differential abundance analysis (DESeq2) revealed unique clustering of significantly differentiated ASVs (adjusted p value < 0.1) across groups with similar functional potential for N and S metabolisms (Fig. 7). Group N1 and S had the greatest number of differentially enriched ASVs (68 and 64 respectively), compared with fewer in groups N2, and N3 (35 and 38 respectively). Putative N functional metabolisms (e.g., aerobic nitrification, denitrification, and dissimilatory N-reduction) are present across all groups, but differ by taxa. Putative S functional metabolisms (e.g., S-oxidation, and S-reduction) are highly enriched in groups S and N1, dominated by a putative S-oxidizing, N-reducer (*Acinetobacter*) in group S. A summary of the significantly different ASVs with the highest enrichment (mean fold change) organized by N and S metabolism is discussed in Supplementary Information and Supplementary Table 1. Results presented here are focused on the dominant putative S-oxidizing, N-reducing taxa differentiated across PCA groups.

Putative S-oxidation was highly enriched in group S (27 ASVs) and N1 (20 ASVs), dominated by taxa capable of both S-oxidation, and N-reducing taxa (Fig. 7; Supplementary Table 1). The highest enriched ASV in group S belonged to a putative S-oxidizing and N-reducer, genus *Acinetobacter* (12 ASVs). However, another putative S-oxidizing, N-reducer, *Thiobacillus* (3 ASVs) had the highest enrichment across all taxa in group N1. Group N2 taxa was highly enriched in *Acinetobacter*, but also *Pseudomonas*, while group N3 had unique contributions from *Rhodobacter*, and *Dechloromonas*. In addition, taxa capable of putative S-oxidation not coupled with N reduction was diverse across groups, with unique contributions in groups N1, S and N2. Group S had contributions from *Sulfuricurvum* (2 ASVs) and *Methylobacterium-Methylorubrum* (3 ASVs), compared to putative S-oxidizers in group N1 including *Sulfurifustis* (2 ASVs), *Magnetovibrio* (1 ASV), and *Meiothermus* (1 ASV) and contributions from *Chromatiaceae* (1 ASV), and *Chlorobium* (1 ASV) in group N2.

## Discussion

### Groundwater sulfur geochemistry driven by geothermal and microbial activity

Groundwater samples from group S, which are located near the northwest rift zone (Fig. 1), were biogeochemically unique and characterized by high SO_4_^2−^. Samples from group S have a distinct volcanic CO_2_ signal suggesting geothermal outgassing [18], which can include sulfur gasses (e.g., H_2_S, SO_2_^2−^) [76], as well as elevated concentrations of PO_4_^3−^ and Si as in our samples, indicating increased weathering of rock minerals. Groundwater age dating from the rift zone (C^14^) indicates much older water (>5000 years) due to either excess geothermal-derived inorganic C, or isolated flow paths resulting in decreased connectivity with the rest of the aquifer [77, 78]. Decreased recharge, and older age indicates a diminished likelihood of abiotic production of oxidized S species such as SO_4_^2−^, and S_2_O_3_^2−^ from volcanic outgassing. However, biotic H_2_S oxidation rates have been shown to far exceed abiotic oxidation in both aerobic and anaerobic conditions [36], further suggesting that SO_4_^2−^ production in these samples may be produced by microbial activity.

Multivariate analysis demonstrated that SO_4_^2−^ concentrations are significant drivers of microbial community structure in both groups S and N1 (Fig. 4 CCA), consistent with other groundwater microbiome studies [5]. The S group was dominated by a high diversity (45 of the 159 ASVs) of putative S-oxidizers belonging to the genus *Acinetobacter*. The genus has been characterized in oligotrophic groundwater with a versatile genome and a great potential for water treatment [79, 80]. FAPROTAX classified *Acinetobacter* as a N-reducer [66], however more recent work has shown members have capabilities for nitrification (oxidation of ammonia to nitrate) using organic substrates (heterotrophic) and aerobic denitrification [80]. Several species of *Acinetobacter* have been described in several industrial systems including wastewater treatment of sulfur, Mn [81], and H_2_ [79]. *Acinetobacter* strains were first identified in the process of denitrifying sulfide removal using microbial communities capable of both heterotrophy and autotrophy to gain energy from S-oxidation with a complete set of genes to reduce NO_3_^2−^ to N_2_ [81].

Taxa classified to *Acinetobacter* were 4-times more enriched in group S than any other N groups. The presence of *Acinetobacter* in our samples may indicate a direct linking of S and N functional metabolisms [82, 83], which is also supported by high *nirS* gene abundances throughout our samples. *Acinetobacter* contains one copy of the *nirS* gene [80] and high *nirS* relative abundances suggests that there is great potential for microbially-driven S-oxidation and N-removal in group S. This study identifies the functional capacity in Hawaiian volcanic aquifers for an *Acinetobacter*-type groundwater microbial community that has the potential to utilize energy from S to drive complete N removal. *Acinetobacter* has demonstrated high N removal efficiency under aerobic conditions (>2 mg L^−1^ DO) in low carbon, low temperature systems where it can remove up to 40.2% of N as gas at a rate of 0.203 mg L^−1^ h^−1^ [84]. The high sulfate concentrations coupled with significantly lower NO_x_^−^ in group S compared to groups N2 and N3, further supports our hypothesis that the electrons required for N-reduction originates from microbial oxidation of sulfur producing SO_4_^2−^ by *Acinetobacter* (S group) and possibly *Thiobacillus* (N1 group).

### Land management significantly influences groundwater quality and microbial N cycling

Land management influences the N geochemistry in the Hualālai watershed primarily from impacts of urbanization (OSDS quantity) in groups N2 and N3 (Keauhou). Inputs of N have a major effect on microbial community structure in groundwater, and may differ depending upon the anthropogenic source of the N [6, 23, 85]. In this study, quantities of OSDS are the primary driver of differences (48%; Fig. 2B) and were significantly associated with microbial community structure (Fig. 4). Results demonstrating OSDS as a driver of groundwater geochemistry are consistent with a groundwater flow and nutrient transport model developed for the coastal region of the Keauhou aquifer (N3) [35]. The model shows that OSDS contributed the largest proportion of nutrients (54% of total N) to the aquifer and had the greatest effect on water quality relative to other nonpoint source contaminants [86]. In this study, areas of high urban densities with high OSDS quantities (N2, N3) had significantly higher NO_x_^−^ concentrations than comparable areas dominated by passive agricultural land use (N1).

Human impacts from agriculture and wastewater are important determinants of geochemistry, water quality, and microbial community structure (especially N-cycling functional groups) in groundwater ecosystems [12, 21]. In this study, the highly enriched putative microbial N-removal function is complete denitrification (heterotrophic nitrification coupled with aerobic denitrification) via sulfur oxidation by *Acinetobacter*. We hypothesize a greater potential for microbial-mediated N-removal in group S compared to the N groups due to the presence of S compounds, elevated sulfate concentrations, and highly enriched *Acinetobacter* populations.

The availability of labile organic carbon is thought to limit the viability of heterotrophic N-removal in groundwater [24, 87]. Sewage effluent that is well oxidized also tends to contain smaller amounts of labile carbon [23] limiting microbial N-reduction processes in some environments. The potential for microbial mediated N-reduction is greatest in group N3 compared to group S based on taxa comparisons and abundances (*nirS*), however, group N3 may be limited by electron donor availability which is greater in group S due to the presence of S compounds. Further studies are necessary to delineate the microbial N-reducution capabilities in these groundwater aquifers and the differences that appear across our groups, especially in areas heavily influenced by OSDS as observed in groups N2 and N3.

### Implications for management

Findings for the enriched potential of linked chemolithoautrophic sulfur-oxidizing and denitrification in the Hualālai watershed has implications for water managers. Although denitrification by sulfide oxidation leads to decreased N loading, the metabolism is potentially detrimental to well or pump operations. Sulfate concentrations, water hardness, and corrosion may increase, thereby causing ions to precipitate out of solution when oxygen is encountered as groundwater is pumped to the surface [6, 23]. Personal communication with private well owners along the rift zone confirmed that some wells do experience rancid, foul-smelling water and precipitation. However, the significant presence of *Acinetobacter* infers the bio-remediation potential of volcanic groundwater microbial communities to simultaneously remove N and S providing an ecosystem service for resource-constrained groundwater aquifers.

## Supplementary information

Supplemental Figure 1

Supplemental Figure 2

Supplemental Figure 3

Supplemental Figure 4

Supplemental Figure 5

Supplemental Figure 6

Supplemental Information

Supplemental metadata

## Data Availability

The datasets generated and analyzed during the current study are available at the Hydroshare repository, https://www.hydroshare.org/resource/d812bbb7c93348999371c9f1f517297f/ and the National Center for Biotechnology Information (NCBI) repository, ID PRJNA819449. All further data and analysis discussed in this study is provided in manuscript supplemental information.
