# Supplementary figures and images for: Geology and land use shape nitrogen and sulfur cycling groundwater microbial communities in Pacific Island aquifers
Source: ISME Commun. 2023 Jun 7;3:58. doi: 10.1038/s43705-023-00261-5 (PMC10247779; doi:10.1038/s43705-023-00261-5)

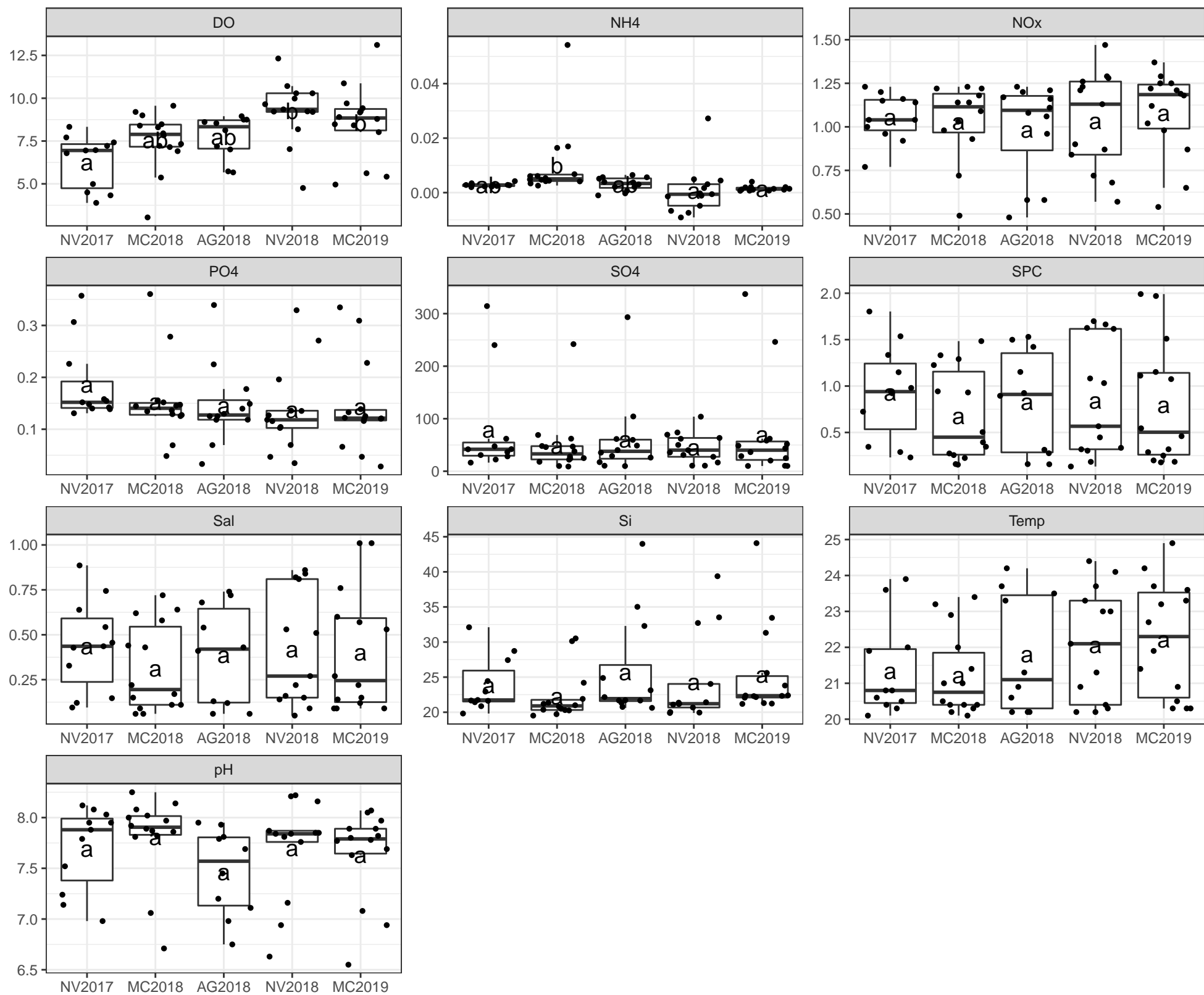

Supplement: Supplementary file 1 — Supplemental Figure 1 [file 43705_2023_261_MOESM1_ESM.pdf]

NMDS by PCA group (PERMANOVA:  $pval=1e-05$ ,  $R^2=0.20$ )

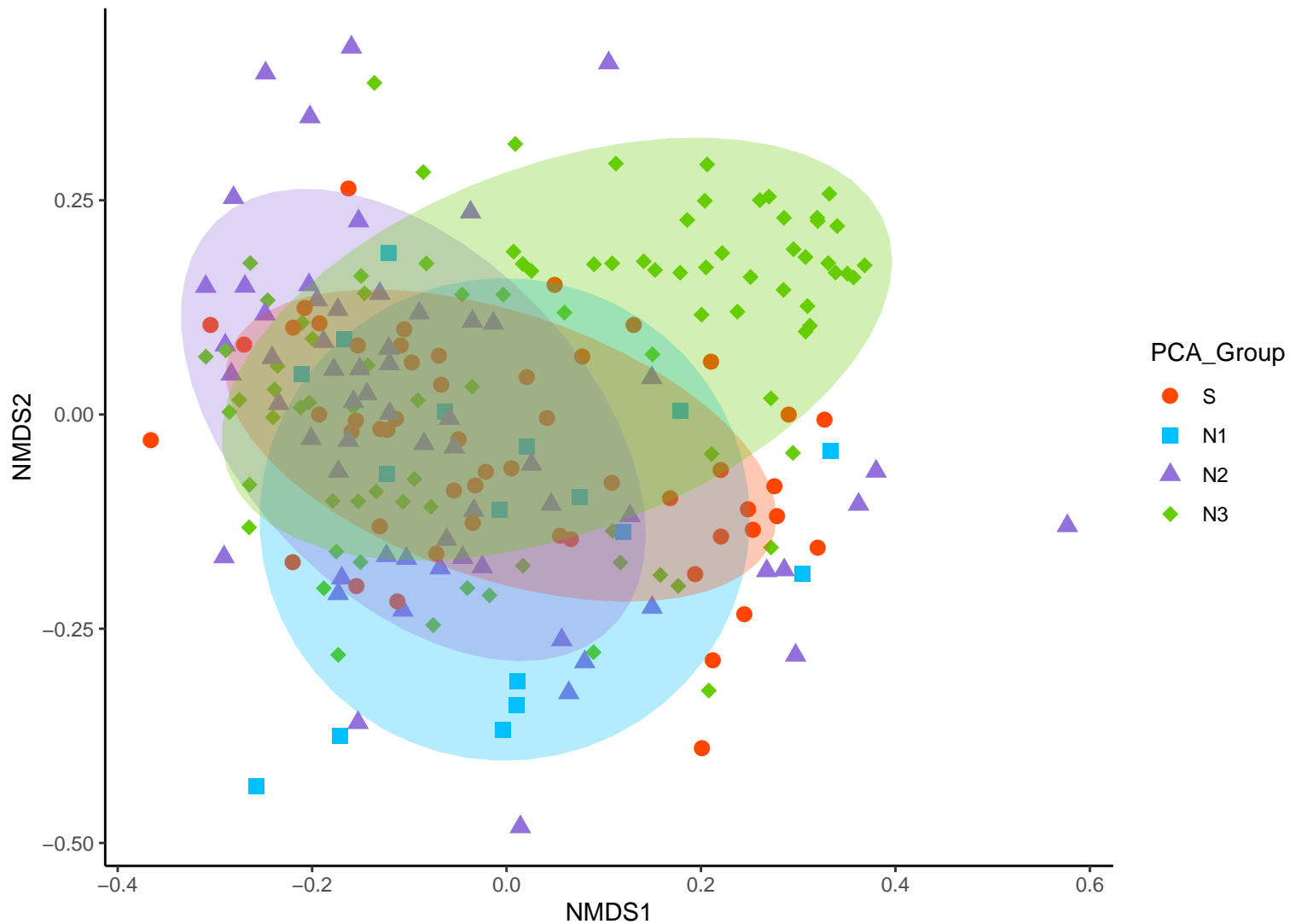

Supplement: Supplementary file 3 — Supplemental Figure 3 [file 43705_2023_261_MOESM3_ESM.pdf]

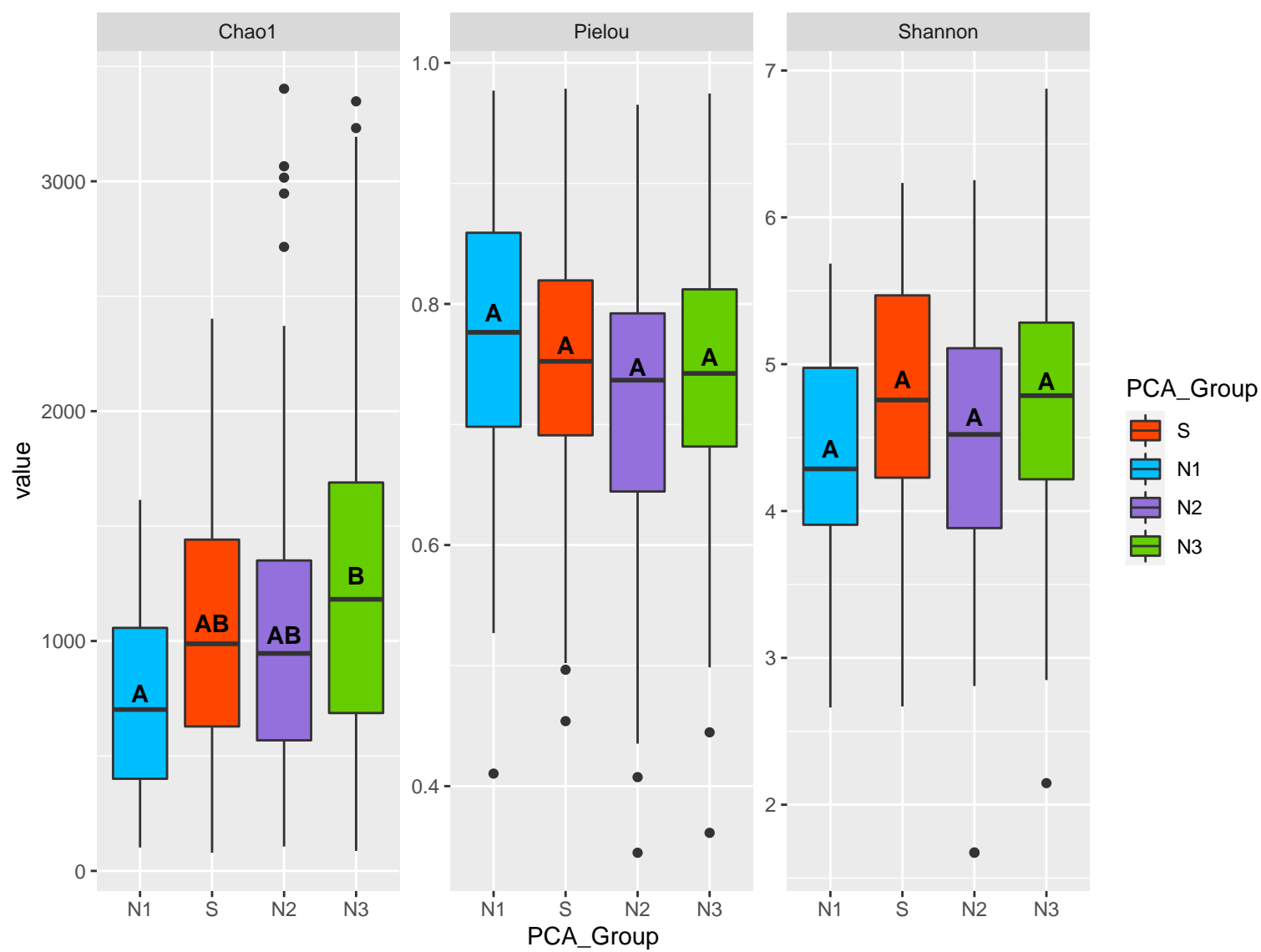

Supplement: Supplementary file 4 — Supplemental Figure 4 [file 43705_2023_261_MOESM4_ESM.pdf]

NMDS by Season (PERMANOVA:  $p\text{val}=0.0001$ ,  $R^2=0.05$ )

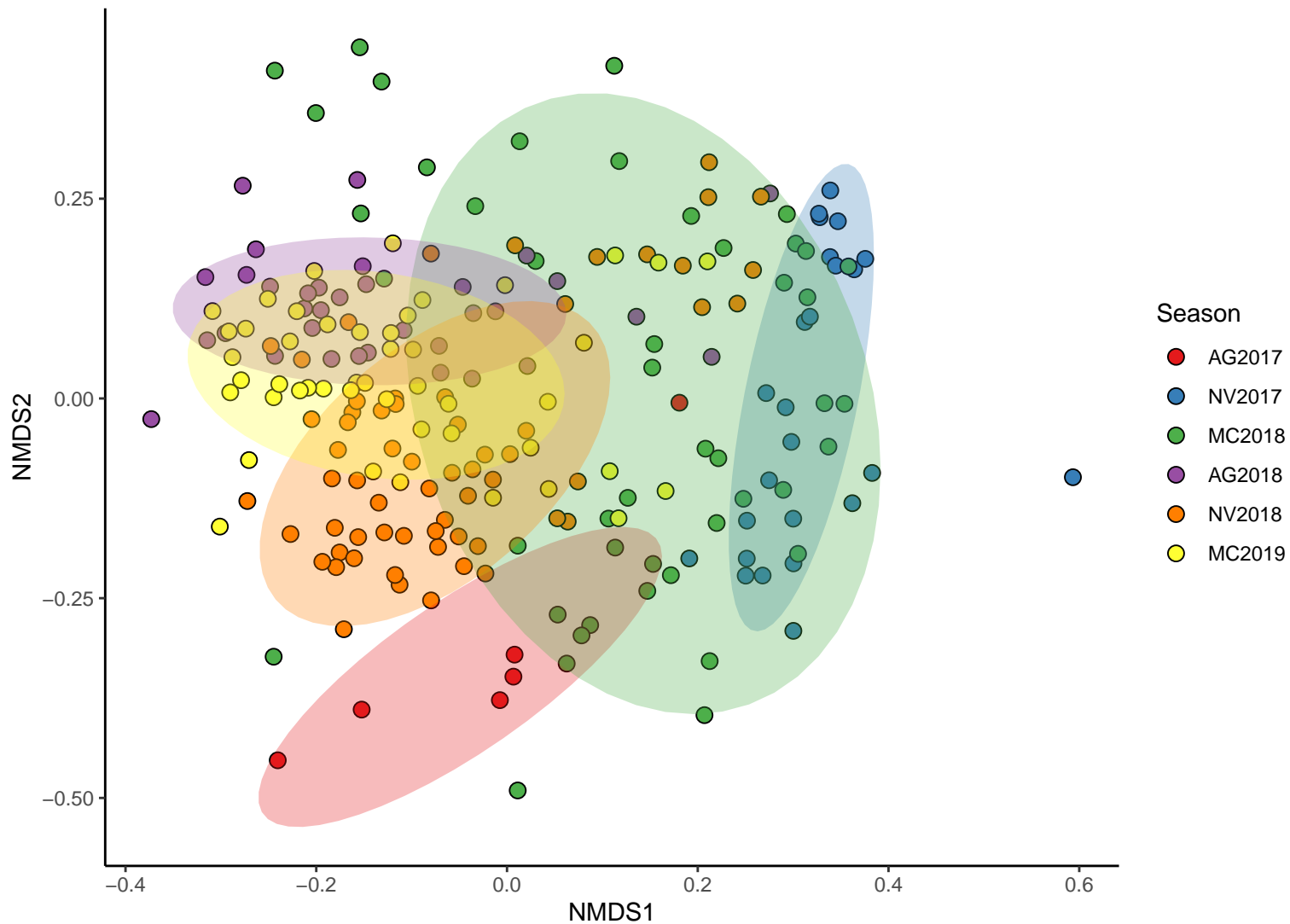

Supplement: Supplementary file 5 — Supplemental Figure 5 [file 43705_2023_261_MOESM5_ESM.pdf]

NMDS by Library (PERMANOVA:  $pval=0.0001$ ,  $R^2=0.06$ )

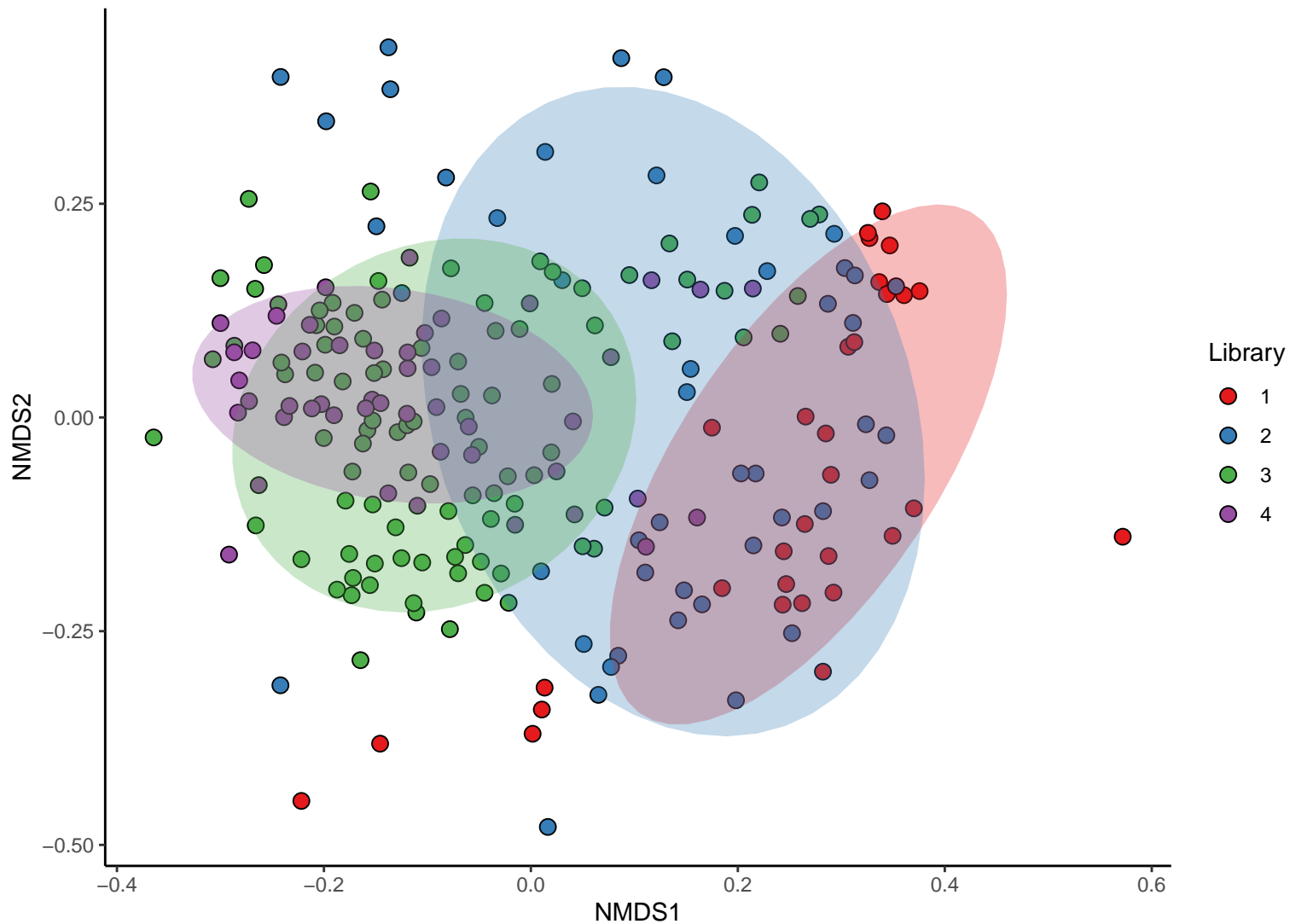

Supplement: Supplementary file 6 — Supplemental Figure 6 [file 43705_2023_261_MOESM6_ESM.pdf]
