# Supplementary material for: Geology and land use shape nitrogen and sulfur cycling groundwater microbial communities in Pacific Island aquifers
Source: ISME Commun. 2023 Jun 7;3:58. doi: 10.1038/s43705-023-00261-5 (PMC10247779; doi:10.1038/s43705-023-00261-5)
Supplement: Supplementary file 2 — Supplemental Figure 2 [file 43705_2023_261_MOESM2_ESM.pdf]

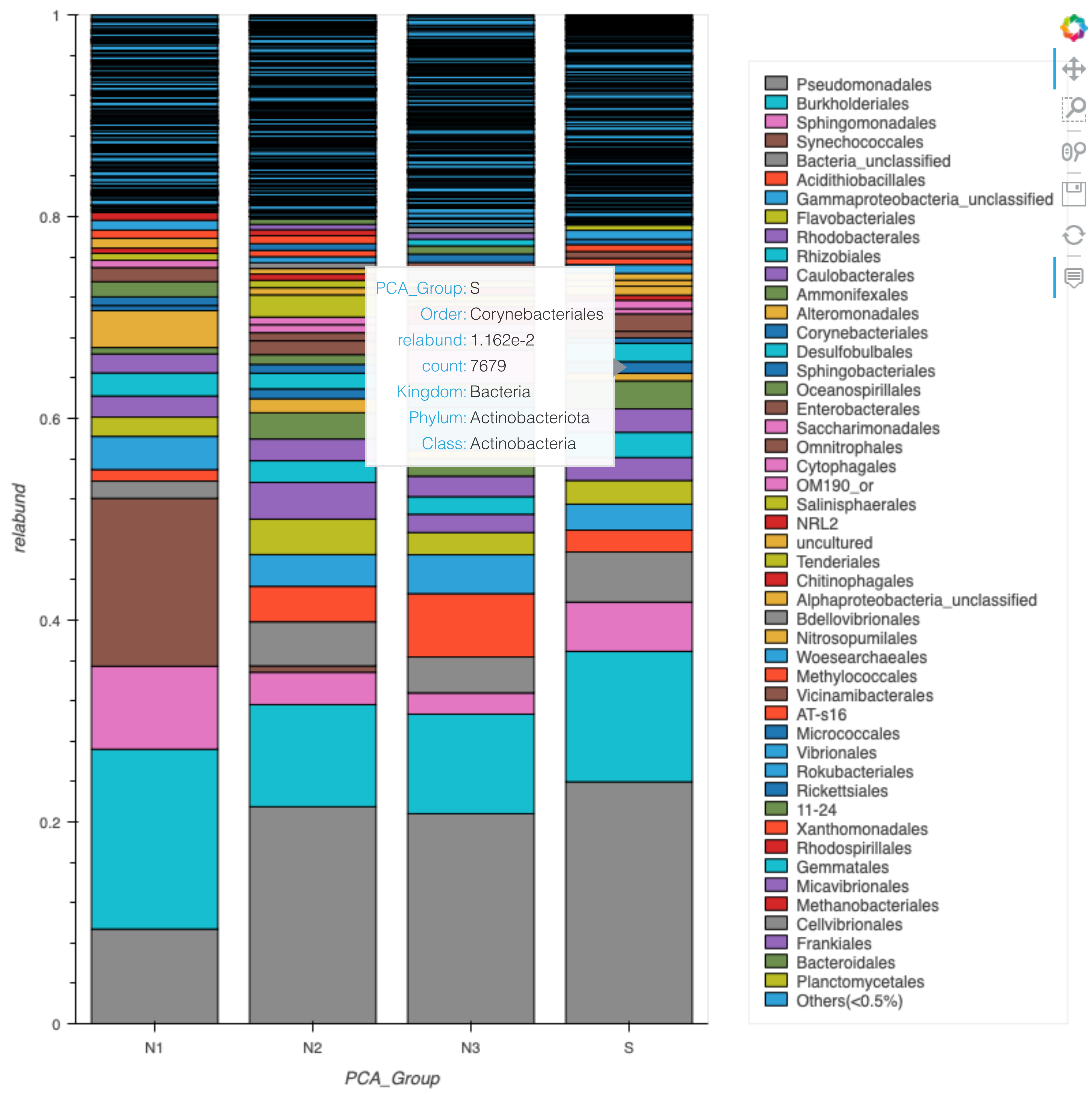

PCA_Group: S
Order: Corynebacteriales
relabund: 1.162e-2
count: 7679
Kingdom: Bacteria
Phylum: Actinobacteriota
Class: Actinobacteria
relabund
0
0.2
0.4
0.6
0.8
1
N1
N2
N3
S
PCA_Group
Pseudomonadales
Burkholderiales
Sphingomonadales
Synechococcales
Bacteria_unclassified
Acidithiobacillales
Gammaproteobacteria_unclassified
Flavobacteriales
Rhodobacterales
Rhizobiales
Caulobacterales
Ammonifexales
Alteromonadales
Corynebacteriales
Desulfobulbales
Sphingobacteriales
Oceanospirillales
Enterobacterales
Saccharimonadales
Omnitrophales
Cytophagales
OM190_or
Salinisphaerales
NRL2
uncultured
Tenderiales
Chitinophagales
Alphaproteobacteria_unclassified
Bdellovibrionales
Nitrosopumilales
Woesearchaeales
Methylococcales
Vicinamibacterales
AT-s16
Micrococcales
Vibrionales
Rokubacteriales
Rickettsiales
11-24
Xanthomonadales
Rhodospirillales
Gemmatales
Micavibrionales
Methanobacteriales
Cellvibrionales
Frankiales
Bacteroidales
Planctomycetales
Others(<0.5%)
