# Supplementary material for: Geology and land use shape nitrogen and sulfur cycling groundwater microbial communities in Pacific Island aquifers
Source: ISME Commun. 2023 Jun 7;3:58. doi: 10.1038/s43705-023-00261-5 (PMC10247779; doi:10.1038/s43705-023-00261-5)
Supplement: Supplementary file 7 — Supplemental Information [file 43705_2023_261_MOESM7_ESM.docx]

**Materials and Methods**

Geochemical Analysis

Total N and P was analyzed as PO_4_^3-^ and NO_x_^-^ following alkaline persulfate digestion (1). Major ions were analyzed using a dual Dionex ICS-1100 Ion Chromatograph following the US EPA Method 300.1 for determining inorganic ions in drinking water (2). Trace metals were analyzed using a Thermo Scientific iCAP 6300 Duo inductively coupled plasma optical emission spectrometer (ICP OES) following EPA 200.7 method (3).

**DNA extraction and 16S rRNA Illumina sequencing**

The 16S rRNA gene sequences were amplified using dual-indexed primers for the V4 region (forward - 515F, 5’-GTGCCAGCMGCCGCGGTAA-3’; reverse - 806R, 5’-GGACTACHVGGGTATCTAAT-3’). PCR amplification was performed in 96 well plates in a 20µL total reaction volume and contained 15µL of Accuprime Master Mix (Invitrogen, Carlsbad, CA), 3µL of genomic DNA, and 1µL of each primer (0.5µM). PCR conditions included an initial denaturation step at 94℃ for 3 minutes followed by 35 cycles of 94℃ for 45s, 50℃ annealing for 60s, and 72℃ for 90s followed by an elongation step for 10min at 72℃ [(3)](https://paperpile.com/c/dZ3cTL/YrJ5). Libraries were normalized with a SequalPrep kit (Invitrogen, CA) and purified using a QIAquick PCR purification kit (QIAGEN Sciences, MD) to a concentration of ~10nM, and then quantified with a Qubit fluorometer (Invitrogen, CA). Sequencing was performed on an Illumina MiSeq (300 cycle, V3 chemistry kit) at the UCI Genomics High-Throughput Facility, at U of California, Irvine.

Quantitative PCR (qPCR) analysis

Quantitative PCR was performed on a StepOnePlus real time machine (Applied Biosystems, Carlsbad, CA) using Applied Biosystems (AB) SYBR green Mastermix (Warrington, UK) in a total reaction volume of 20µL. Reactions were performed in triplicate with 2µL of genomic DNA template, primers at 0.5µM for 16S and nitrite reduction (*nirS)*, and 1.0µM for sulfate reduction (*dsrA)* in 96-well plates sealed with adhesive optical covers (SI). Stock gBlock standards (Integrated DNA Technologies, Coralville, IA) for qPCR were diluted to a concentration of 1x10^9^ copies µL^-1^. Seven-point calibration curves were created by a 10-fold serial dilution in triplicate for each qPCR reaction ranging from 10^2^ to 10^8^ target copies. Detection limits ranged from 10^2^ to 10^4^ copies and blanks were below detection limits (SI). Abundance calculations were made by summing fractionated samples (0.8 + 0.2 µm).

**Results**

Drivers of microbial diversity

Community composition also differed significantly by season (PERMANOVA, p<0.001; Supp Fig.5). However, because libraries were prepped and sequenced in chronological order, we cannot distinguish library batch effects from effects due to seasonality as community composition also significantly differed by sequence run (PERMANOVA; p<0.001; SI Fig.6). Therefore, the temporal variability of these communities is not included in the discussion.

Differential abundance analysis (DESeq2)

Putative S-reduction has the most enriched (19) ASVs in group N1 with a high diversity of different taxa across groups (SI Table 1). Putative S-reduction in group N1 (15 ASVs) included highly enriched uncultured members of *Desulfobacterota* (2 ASVs), *Geobacteraceae* (3 ASVs), and *Desulfovibrio* (2 ASVs), compared to only one enriched unclassified member of Desulfobulbales (1 ASV) in group S. Group N2 had unique putative S-reducing ASVs including highly enriched members of *Shewenella* (2 ASVs), and *Alishewanella* (2 ASVs). Taxa belonging to unclassified members of *Desulfobacterota* (2 ASVs), and Alishewanella (2 ASVs) were also highly enriched in group N3.

Putative aerobic nitrifiers were mostly Archaea, but differed by taxa across groups (SI Table 1). Archaea (11 out of 20) is the largest enrichment in group N1 in unclassified members of *Nitrosopumilaceae*, while the largest bacterial enrichment includes members of genus *Nitrospira*. In group S, putative aerobic nitrifiers were also predominantly Archaea (13 out of 19), the greatest enrichment belonging to genus *Nitrosarchaeum*, and contributions from bacteria classified to genus *Candidatus* Nitrotoga. Groups N2 (6 ASVs) and N3 (8 ASVs) had less than half the enriched ASVS classified as putative aerobic nitrifiers compared to groups N1 and S. All putative aerobic nitrifiers in group N2 belonged to Archaea with the largest enrichment from uncultured genus *Nitrososphaeria*. Group N3 was also dominated by Archaea (7 out of 8) with the greatest enrichment in genus *Candidatus* Nitrosotenuis, and the lone bacteria ASV belonging to genus *Nitrospira* similar to group N1.

Putative denitrifiers and dissimilatory N-reducers belonged primarily to *Flavobacterium* across groups (SI Table 1). Group N3 had the greatest number of ASVs (11), with the largest enrichment and the largest number (7 out of 11) classified as genus *Flavobacterium*. This was similar in group N2, as *Flavobacterium* made up most of the taxa (9 out of 10 ASVs) and highest enrichment. There were fewer total ASVs (7) in group S, but also predominantly classified to *Flavobacterium* (5 out of 7). Two unique ASVs in group S belonged to *Denitratisoma* and *Bradyrhizobium*. Group N1 taxa (6 ASVs) are very different from groups S, N2 and N3 as there were no contributions from *Flavobacterium*, but rather included unique genera *Nocardioides*, *Marinobacter*, and *Hyphomonas*.

Putative dissimilatory N-reduction is highly enriched (11) in group N1 with similar taxa occurring across all groups. Group N1 has the greatest number of classified ASVs (3) belonging to genus *Anaeromyxobacter* with the largest enrichment. In addition, two other ASVs in group N1 had high enrichments including genus *Streptomyces* and genus *Thalassospira* largely enriched in both groups N1 and S. Group S is different (7 ASVs) with the highest enrichment classified to genus *Aquabacterium* (2 ASVs), and *Allorhizobium* (1 ASV). Fewer enriched taxa occurred in both N2 and N3 (5), the highest enriched ASV belonged to *Aquabacterium* similar to group S. The highest enriched ASV in group N2 classified to *Stenotrophomonas.*

**References**

1. [Methods of analysis by the U.S. Geological Survey National Water Quality Laboratory : evaluation of alkaline persulfate digestion as an alternative to Kjeldahl digestion for determination of total and dissolved nitrogen and phosphorus in water. 2003. Available from:](http://paperpile.com/b/hhpsWQ/jhiIe)<http://dx.doi.org/10.3133/wri034174>.

2. [Hautman DP, Munch DJ. Development of U.S. EPA Method 551.1. Journal of Chromatographic Science 1997;35;p. 221–31.](http://paperpile.com/b/hhpsWQ/Nwtg0) [doi.org/10.1093/chromsci/35.5.221](http://dx.doi.org/10.1093/chromsci/35.5.221).

3. [Caporaso JG, Lauber CL, Walters WA, Berg-Lyons D, Huntley J, Fierer N, et al. Ultra-high-throughput microbial community analysis on the Illumina HiSeq and MiSeq platforms. ISME J. 2012 Aug;6(8):1621–4.](http://paperpile.com/b/dZ3cTL/YrJ5)

4. [Laboratory EMS, Environmental Monitoring Systems Laboratory. Determination of metals and trace elements in water and wastes by inductively coupled plasma-atomic emission spectrometry. In Methods for the Determination of Metals in Environmental Samples. 1996. p.31–87.](http://paperpile.com/b/hhpsWQ/wavr0) [doi.org/10.1016/b978-0-8155-1398-8.50010-0](http://dx.doi.org/10.1016/b978-0-8155-1398-8.50010-0).

**Supplementary Figure and Table Legends**

SI Figure 1. Tukey-style box plots of measured groundwater geochemistry organized by sample time. Vertical y-axis for DO, NH_4_, NO_x_, and SO_4_^-2^ are measured in mg L^-1^, SPC (µS cm^-1^), and Temp is (℃). Post-hoc analysis (Tukey-post hoc) determined significant differences between groups and are labeled by letters to indicate significant difference.

SI Figure 2. Relative abundance (%) of ASVs classified to Order and organized by group (N1, S, N2, N3).

SI Figure 3. Non-metric multidimensional analysis (NMDS) colored by group. PERMANOVA results were significant at the p<0.001 level.

SI Figure 4. Alpha diversity indices including Chao1, Pielou, and Shannon visualized by group. Post-hoc analysis (Tukey-post hoc) determined significant differences between groups and are labeled by letters to indicate significant difference.

SI Figure 5. Non-metric multidimensional analysis (NMDS) colored by sample time (season). PERMANOVA results were significant at the p<0.001 level.

SI Figure 6. Non-metric multidimensional analysis (NMDS) colored by library sequence order. PERMANOVA results were significant at the p<0.001 level.

SI Table 1. A summary of putative N and S cycling ASVs by groups. The symbol A refers to the genus belonging to the Phylum Archaea, and B refers to the genus belonging to Bacteria. The number in parentheses after each genus refers to the mean fold change or enrichment difference between groups.

Supplemental Table 1.

| **Putative N or S Metabolism** | **N1** | **S** | **N2** | **N3** |
| --- | --- | --- | --- | --- |
| **N-cycling** | | | | |
| **Aerobic Nitrifiers** | *Nitrosopumilaceae* (A-6x)  *Nitrospira* (B-7x) | *Nitrosarcheum* (A-4x)  *Candidatus*  *Nitrotoga* (B-7x) | *Nitrososphaeria*  (A-4x)  _______ | *Candidatus*  *Nitrosotenuis*  (A-3x)  *Nitrospira* (B-3x) |
| **Denitrifiers and Dissimilatory N-reduction** | *Nocardioides* (7x)  *Marinobacter* (5x)  *Hyphomonas* (4x) | *Flavobacterium*  (4x)  *Denitratisoma* (6x)  *Bradyrhizobium* (3x) | *Flavobacterium* (3x) | *Flavobacterium* (4x) |
| **Dissimilatory**  **N-reduction** | *Aneromyxobacter*  (3x)  *Streptomyces* (3x)  *Thalassospira* (8x) | *Aquabacterium*  (3x)  *Allorhizobium* (2x)  *Thalassospira*  (8x) | *Stenotrophomonas*  (3x) | *Aquabacterium* (3x) |
| **S-cycling** | | | | |
| **S-oxidation**  **and N-reduction** | *Thiobacillus*  (110x) | *Acinetobacter*  (4x) | *Pseudomonas*  (3x)  *Acinetobacter*  (6x) | *Rhodobacter*  (3x)  *Dechloromonas*  (3x)  *Pseudomonas*  (5x) |
| **S-oxidation** | *Sulfurifustis*(5x)  *Magnetovibrio*(4x)  *Meiothermus*(3x) | *Sulfuricurvum*  (3x)  *Methylobacterium-Methylorubrum* (2x) | *Chromatiaceae*  (3x)  *Chlorobium*  (3x) | _______ |
| **S-reduction** | *Desulfobacterota*  (5x)  *Geobacteraceae*  (4x)  *Desulfovibrio*(4x) | Unclassified  *Desulfobulbales*  (6x) | *Shewenella*(3x)  *Alishewanella*  (3x) | *Desulfobacterota*  (4x)  *Alishewanella* (3x) |
